# The Effect of A2E on the Uptake and Release of Calcium in the Lysosomes and Mitochondria of Human RPE Cells Exposed to Blue Light

**DOI:** 10.1155/2021/5586659

**Published:** 2021-09-23

**Authors:** Mao-Mei Luo, Lin Chen, Shu Wang, Chun Zeng, De-Zhi Li, YeGe Bi, Long-Qian Liu, Shan-Jun Cai

**Affiliations:** ^1^Department of Ophthalmology, Affiliated Hospital of Zunyi Medical University, Zunyi, Guizhou, China; ^2^Key Laboratory of Eye Diseases of Guizhou Province, Guizhou Eye Hospital, Zunyi, Guizhou, China; ^3^Department of Ophthalmology, Dazhou Central Hospital, Dazhou, Sichuan, China; ^4^Department of Ophthalmology, West China Hospital, Sichuan University, Chengdu, Sichuan, China; ^5^Department of Optometry and Visual Science, West China Hospital, Sichuan University, Chengdu, Sichuan, China

## Abstract

We aimed to explore the effect of N-retinylidene-N-retinylethanolamine (A2E) on the uptake and release of calcium in lysosomes and mitochondria by establishing a model of human retinal pigment epithelial (RPE) cell injury induced by exposure to blue light. Primary human RPE cells were cultured from passages 4 to 6 and exposed to blue light at an intensity of 2000 ± 500 lux for 6 hours. After blue light exposure, the culture was maintained for 24 hours. A2E at a final concentration of 25 *μ*M was added to the culture 2 hours before light exposure, and nifedipine at a final concentration of 10^−4^ M was added 1 hour before light exposure. The levels of Ca^2+^ in the cytosol (CaTM/2AM), mitochondria (Rhod/2AM), and lysosomes (LysoTracker Red and Fluo-3/AM) were determined. In order to measure the calcium levels in the different organelles, RPE were imaged using a laser scanning confocal microscope. Moreover, changes in the mitochondrial membrane potential were detected by flow cytometry analysis of JC-1-stained cells. The obtained results revealed that blue light illumination increased the calcium fluorescence intensity in the cytoplasm, mitochondria, and lysosomes of human RPE cells when compared with the control cells (*P* < 0.05). After A2E treatment, the fluorescence intensity of the calcium in the cytoplasm was further increased (*P* < 0.05), while that in the mitochondria and lysosomes decreased (*P* < 0.05). In addition, we observed that nifedipine reduced the fluorescence intensity of calcium in the RPE cells. Our results also showed that the mitochondrial membrane potential in the RPE treated with blue light and A2E was lower than that in the control, blue light, and A2E-treated cells (*P* < 0.05). Blue light increased calcium levels in the cytoplasm, lysosomes, and mitochondria of RPE cells. A2E damages the lysosomal and mitochondrial membranes, resulting in calcium release into the cytoplasm. Finally, our results demonstrated that both blue light and A2E treatments reduced mitochondrial membrane potential, increasing cytosolic Ca^2+^ levels, which can contribute to the activation of RPE death.

## 1. Introduction

Retinal pigment epithelial (RPE) cells have many important functions, including phagocytosis of the photoreceptor outer segments and the undigested cargo, after forming lipofuscin. The accumulation of lipofuscin in RPE cells increases slowly with age, which enhances the sensitivity to light radiation by inducing changes at structural and functional cellular levels. The accumulation of this pigment is related to the increase in the incidence of diseases such as age-related macular degeneration (AMD) and Stargardt disease [1, 2]. As the main component of lipofuscin, N-retinylidene-N-retinylethanolamine (A2E) is fluorescent and phototoxic and is not easily degraded in lysosomes [3]. In the visible light spectrum, blue light is the most harmful, with a wavelength of 400–500 nm that can directly penetrate the lens and reach the retina. Long-term exposure can cause damage to the macula [4]. Under blue light irradiation, the structure of A2E changes and produces superoxide anions, singlet oxygen, and other substances, which reduce the activity of lysosomal enzymes, destroy lysosomal membranes, and target mitochondria, decreasing RPE cell viability and inducing apoptosis [5, 6].

As a second messenger in the cell, calcium (Ca^2+^) can initiate a series of biochemical reactions and is stored in organelles such as lysosomes and mitochondria [7]. The dynamic balance of calcium ions is essential to maintain the function of cells. Sustained high concentrations of Ca^2+^ in cells generate a large amount of reactive oxygen species (ROS), which damage organelle structures, activate various enzymes, and induce apoptosis [8]. Lysosomes and mitochondria can rapidly, reversibly, and selectively absorb the Ca^2+^ released from the endoplasmic reticulum to maintain intracellular Ca^2+^ homeostasis [9]. The Ca^2+^ channel of the lysosome contributes to the generation of Ca^2+^ signals in the cell and regulates the material transportation, fusion, and exocytosis of the lysosome. Ca^2+^ in mitochondria is involved in oxidative phosphorylation, mitochondrial motility, ATP synthesis, and apoptosis of cells. Ca^2+^ overload in mitochondria will open the mitochondrial permeability transport pore, leading to mitochondrial swelling and outer membrane rupture and finally inducing cell apoptosis. Therefore, there is strong interest in the changes in Ca^2+^ levels in lysosomes and mitochondria of RPE cells, under blue light irradiation and A2E loading.

In the visible light spectrum, blue light (BL) is the most harmful, with a wavelength of 400–500 nm that can directly penetrate the lens and reach the retina. Long-term exposure can cause damage to the macula [4]. Early studies of this research group explored the optimal light intensity and time for blue light exposure, finding it to be 2000 ± 500 lux for 6 hours [10], while cultures were maintained in the incubator for 24 hours after blue light exposure. Under this condition, there were more apoptotic cells and fewer necrotic cells. The optimal concentration of A2E was determined to be 25 *µ*M [11, 12]. The cell viability significantly decreased in an A2E-dose-dependent manner, and cell viability was best under this condition. Nifedipine (NFD) was used to eliminate the influence of extracellular Ca^2+^ influx. Using these findings, we explored the effects of A2E on the lysosomal and mitochondrial uptake and release of Ca^2+^ in RPE cells, after blue light irradiation. These results may provide a basis for further elucidation of human RPE cell apoptosis induced by blue light.

## 2. Materials and Methods

### 2.1. Cell Sources and Ethics Statement

Three donor eyeballs were obtained from a healthy 47-year-old male, 53-year-old male, and 62-year-old female who died in an accident. Within 12 hours of death, the eyes were taken after keratoplasty at the Affiliated Hospital of Zunyi Medical University. All experiments adhered to the ethical standards established by the unit's Human Test Committee.

### 2.2. Reagents and Instruments

Dulbecco's modified Eagle's medium (DMEM) and trypsin-EDTA solution were obtained from HyClone (Logan, Utah, USA). Fetal bovine serum (FBS) was obtained from MRC (New Zealand). Hank's balanced salt solution (HBSS), Rhod-2/AM, Fluo-3/AM, and LysoTracker Red DND-99 were obtained from Thermo Fisher Scientific (Waltham, MA). CaTM-2/AM was obtained from Goryo (Sapporo, Japan). NFD and F-127 were obtained from Sigma-Aldrich (St. Louis, MO). Mouse anti-human keratin antibody, goat anti-mouse IgG-HRP, mitochondrial membrane potential assay kit with JC-1, phosphate-buffered saline (PBS), and penicillin-streptomycin were obtained from Solarbio (Beijing, China). Dimethyl sulfoxide (DMSO) was obtained from MP Bio (California, USA). A2E was donated by Sun Xiaodong (The First Affiliated Hospital of Shanghai Jiaotong University).

### 2.3. Primary Culture of Human RPE Cells

Briefly, the eyeballs were cut behind the serrated margin. The cornea, lens, vitreous body, and retinal neuroepithelium were carefully removed to form an eyecup and then washed with HBSS. Approximately 2/3 of the eyecup was filled with 0.25% trypsin and incubated at 37°C for 45 min. The cell suspension was collected and the precipitate was resuspended in DMEM containing 10% FBS, seeded in a 25 cm^2^ cell culture flask, and cultured in an incubator. Cells from the same passage were used for each experiment.

### 2.4. Immunohistochemistry

Established third-generation human RPE cells were seeded on a sterilized cover glass and fixed with 4% paraformaldehyde for 10 min. Then, 3% hydrogen peroxide was added for a 15 min incubation, and cells were permeabilized with 1% Triton X-100 for 30 min at room temperature and washed with PBS. Then, the cells were blocked by goat serum for 10 min and incubated at 4°C overnight with mouse anti-human RPE65 antibody. After incubation in goat anti-mouse IgG antibody for 1 h at 37°C in the dark, nuclei were stained with 4′,6-diamidino-2-phenylindole (DAPI). The cells were visualized by fluorescence microscopy.

### 2.5. Calcium in the Cytoplasm

Fourth-generation human RPE cells were seeded in confocal dishes at a density of 1 × 10^5^ cells/ml and divided into five groups: control cells (control), blue light-treated cells (BL), blue light + NFD-treated cells (BL + NFD), A2E + blue-treated cells (A2E + BL), and A2E + blue light + NFD-treated cells (A2E + BL + NFD). A2E at a final concentration of 25 *μ*m was added to cells in the dark 2 hours before they were illuminated, and NFD at a final concentration of 10^−4^ M was added 1 hour before they were illuminated. Then, the cells were exposed to blue light in an incubator for 6 hours (the cells in the control group were wrapped in tin foil) with an intensity of 2000 ± 500 lux, and the culture was maintained for 24 hours after illumination.

CaTM-2/AM was dissolved in anhydrous DMSO to a concentration of 4 mM in the dark and then diluted with HBSS to a final concentration of 8 *μ*M, and F-127 with a mass fraction of 20% was added. The CaTM-2/AM working solution was added to the cells in the dark and the cells were incubated at 37°C for 40 min. Then, the cells were washed twice and incubated in HBSS at 37°C for another 20 min. Then, a laser scanning confocal microscope was used to observe and take photos. The excitation wavelength was 561 nm. The fluorescence intensity indicated the number of calcium ions, and ImageJ was used for the analysis.

### 2.6. Calcium in Mitochondria

The cell groupings and pretreatment were the same as those described in Section 2.5. Rhod-2/AM was dissolved in anhydrous DMSO to a concentration of 5 mM in the dark, HBSS was added to dilute the dye to a final concentration of 5 *μ*M, and F-127 with a mass fraction of 20% was added. The pretreated cells in each group were loaded with Rhod-2/AM working solution and incubated at 37°C for 30 min in the dark. Then, the cells were washed twice with HBSS and left for a further 20 min at 37°C. A laser scanning confocal microscope was used to observe and take photos. The excitation wavelength was 561 nm. ImageJ was used to analyze the fluorescence intensity, which represented the number of calcium ions.

### 2.7. Calcium in Lysosomes

The experimental groups and pretreatment were the same as those described in Section 2.5. Fluo-3/AM was dissolved in anhydrous DMSO in the dark at a concentration of 5 mM and diluted with HBSS to a final concentration of 10 *μ*M; then, F-127 with a mass fraction of 20% was added. The pretreated cells were loaded with Fluo-3/AM working solution and incubated at 37°C in the dark for 30 min. Then, the cells were washed with HBSS twice and incubated at 37°C for 20 min to allow the complete conversion of Fluo-3/AM to Fluo-3. Then, HBSS was aspirated by pipette, and LysoTracker Red at a final concentration of 20 nM was added to the cells in the dark and left for a further 10 min at 37°C. Then, a laser confocal microscope was used to observe the cells after Fluo-3 was stimulated at an excitation wavelength of 488 nm and LysoTracker Red was stimulated at an excitation wavelength of 561 nm. Lysosomal fluorescence and intracellular calcium ion fluorescence were colocalized to detect calcium in the lysosome, and the fluorescence intensity was used to represent the calcium ion levels. ImageJ was used for analysis.

### 2.8. Mitochondrial Membrane Potential

Fourth-generation human RPE cells were divided into four groups: control-treated cells (control), blue light-treated cells (BL), A2E-treated cells (A2E), and A2E + blue-treated cells (A2E + BL). The concentration of A2E and the measurement parameters in terms of time and intensity of light were the same as those described in Section 2.5.

After pretreatment, the cells were digested with 0.25% trypsin and the cell suspension was collected and centrifuged to remove the supernatant, followed by the addition of JC-1 dye (working concentration: 10 *μ*g/mL, ultrapure water dilution), and the cells were incubated at 37°C for 20 min. The cell populations were assessed by flow cytometry. The number of cells recorded in each experimental group was ≥10000.

### 2.9. Image Process Analysis

For quantitative analysis of the fluorescence of calcium, the experimental results of each part were maintained at the same threshold by ImageJ, so as to select the region of interest and analyze the average fluorescence intensity.

### 2.10. Statistical Analysis

The results are expressed as the mean ± SD and were analyzed with SPSS 18.0 statistical software. One-way analysis of variance (ANOVA) followed by an LSD test was performed to compare differences for multiple groups. Differences were considered significant when *P* < 0.05.

## 3. Results

### 3.1. Human RPE Cells and Immunohistochemistry

**Figure 1:**
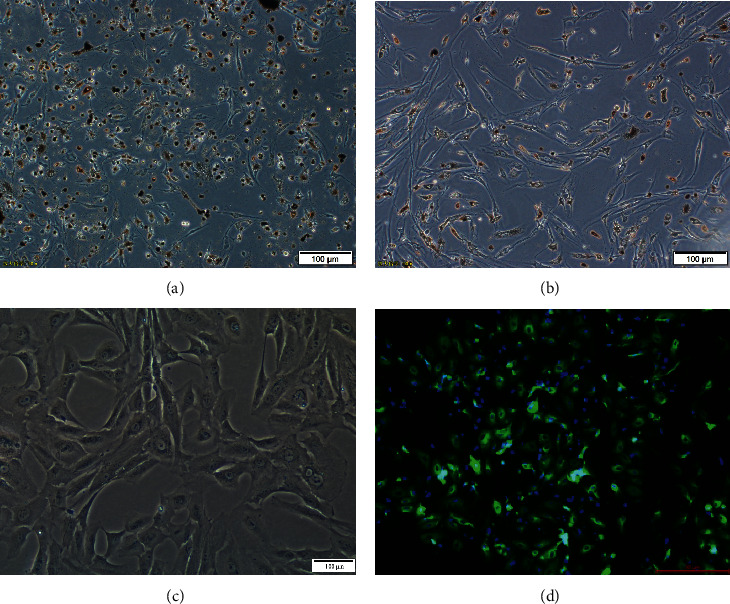
Human RPE cells and immunohistochemistry. (a) After being cultured for 3 days, primary human RPE cells were spindle-shaped or polygonal and the cytoplasm was filled with pigment particles. (b) Seven days later, the cells were spindle-shaped or irregular, with round and binuclear nuclei. (c) The third generation of human RPE cells had fewer pigment particles in the cytoplasm and presented as spindles or irregular polygons. (d) Primary mouse anti-human RPE65 antibodies and secondary goat anti-mouse IgG antibodies were used to immunostain third-generation RPE cells. Immunofluorescence staining was positive for RPE65.

Primary human RPE cells adhered to the dish wall. After 3 days in culture, the cells were spindle-shaped or polygonal, and the cytoplasm was enriched with brown-black particles. Seven days later, the cells showed pseudopods, and transparent round nuclei appeared, some of which were binuclear. As the number of passages increased, more RPE cells were spindles or irregular polygons, and the number of pigment particles in the cytoplasm gradually decreased. Immunofluorescence staining showed green fluorescence in RPE cells, indicating expression of RPE65 (Figure 1).

### 3.2. Calcium Levels in the Cytoplasm of RPE Cells

**Figure 2:**
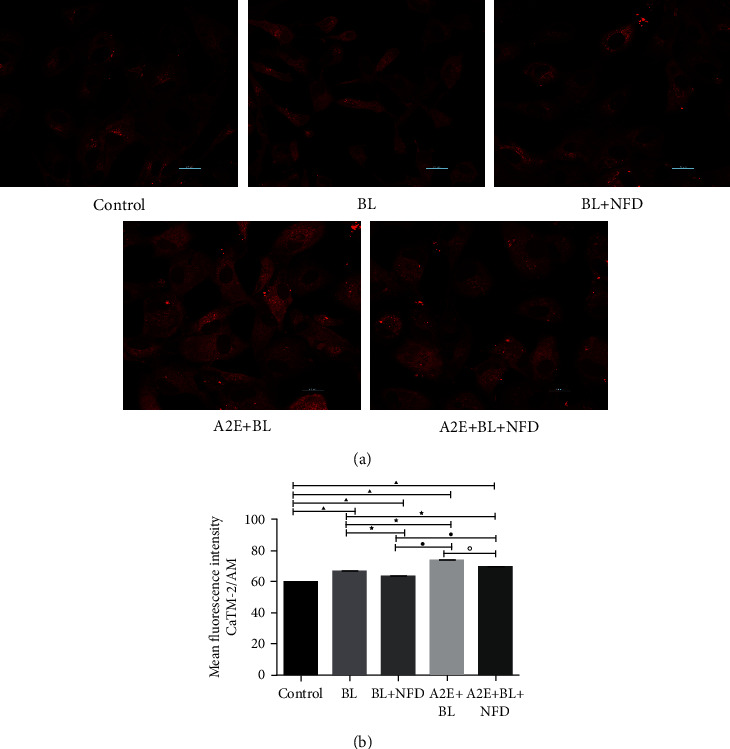
The level of calcium in the cytoplasm. (a) To investigate the Ca^2+^ level in the cytoplasm, we performed CaTM-2/AM staining. Our data showed that blue light and A2E significantly elevated the cytoplasmic Ca^2+^ levels compared with those in the nontreated cells. Scale bar = 20 *μ*M. (b) Data are presented as the mean ± SD (*n* = 3). An LSD test following one-way ANOVA was performed for the statistical analysis. ▲*P* < 0.05, compared with the controls; ★*P* < 0.05, compared with the BL group; ●*P* < 0.05, compared with the BL + NFD group; and ◇*P* < 0.05, compared with the A2E group.

To investigate the Ca^2+^ level in the cytoplasm, we measured the change in intensity of the Ca^2+^-sensitive dye CaTM-2/AM. We found that the Ca^2+^ level in the control cells was lower than that in all the other treated cells (*P* < 0.05). The Ca^2+^ level in the cells treated with BL + NFD was lower than that in the BL-treated cells (*P* < 0.05). The A2E and A2E + NFD-treated cells showed a significant increase in the Ca^2+^ level compared with the BL-treated cells (*P* < 0.05). The Ca^2+^ level in the A2E + BL and A2E + BL + NFD-treated cells was higher than that in the BL + NFD-treated cells (*P* < 0.05). The Ca^2+^ level in the cells treated with A2E + BL was significantly decreased compared with that in the A2E-treated cells (*P* < 0.05) (Figure 2).

### 3.3. Calcium Levels in the Mitochondria of RPE Cells

**Figure 3:**
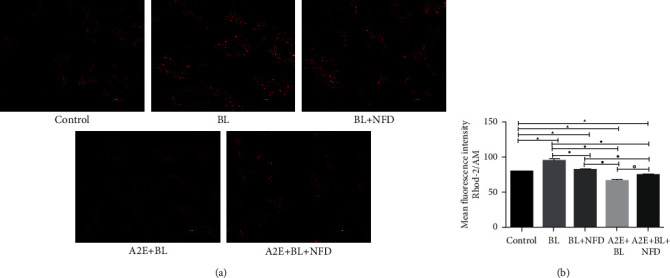
The calcium level in mitochondria. (a) For the determination of Ca^2+^ levels in mitochondria, the fluorescence intensity of calcium in RPE cells was assessed by confocal microscopy using the calcium-sensitive dye Rhod-2/AM. Scale bar = 20 *μ*M. (b) Data are presented as the mean ± SD (*n* = 3). An LSD test following one-way ANOVA was performed for the statistical analysis. ▲*P* < 0.05, compared with the controls; ★*P* < 0.05, compared with the BL group; ●*P* < 0.0505, compared with the BL + NFD group; and ◇*P* < 0.05, compared with the A2E group.

The calcium in the mitochondria was labeled by Rhod-2/AM, an indicator of Ca^2+^, and the fluorescence intensity was measured using a laser scanning confocal microscope. The level of Ca^2+^ in the BL and BL + NFD-treated cells was significantly increased compared with that of the control cells (*P* < 0.05), while a significant decrease was observed in the A2E + BL and A2E + BL + NFD-treated cells compared to the control cells (*P* < 0.05). The BL + NFD, A2E + BL, and A2E + BL + NFD-treated cells presented a decreased levels of Ca^2+^ (*P* < 0.05) compared to BL-treated cells. The Ca^2+^ level in the A2E + BL and A2E + BL + NFD-treated cells was lower than that in the BL + NFD-treated cells (*P* < 0.05). The cells treated with A2E + BL + NFD exhibited an increased level of Ca^2+^ compared with the A2E-treated cells (*P* < 0.05) (Figure 3).

### 3.4. Calcium Levels in the Lysosomes of RPE Cells

**Figure 4:**
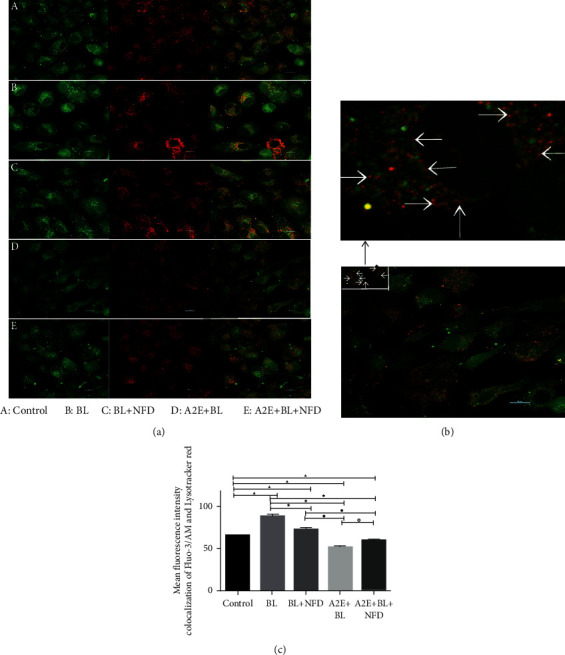
The level of calcium in lysosomes. (a) The green fluorescence is the intracellular Ca^2+^ fluorescence labeled with Fluo-3/AM, the red fluorescence is the lysosome labeled by LysoTracker Red, and the yellow fluorescence is the Ca^2+^ fluorescence in the lysosome after the two fluorescence signals are colocalized. Scale bar = 20 *μ*M. (b) The zoom of colocalization between Fluo-3/AM and LysoTracker Red-treated cultured RPE cells. (c) Data are presented as the mean ± SD (*n* = 3). An LSD test was conducted following one-way ANOVA. Compared to the control group, ▲*P* < 0.05; compared to the BL group, ★*P* < 0.05; compared to the BL + NFD group, ●*P* < 0.05; and compared to the A2E group, ◇*P* < 0.05.

To determine the Ca^2+^ level in lysosomes, we performed Ca^2+^ imaging with Fluo-3/AM and lysosomes with LysoTracker Red using laser confocal microscopy. With these colocalizing dyes, the level of Ca^2+^ in the lysosomes can be analyzed. The lysosomes from BL and BL + NFD-treated cells showed an increase in the Ca^2+^ level compared with that of the control cells (*P* < 0.05). Moreover, cells treated with A2E and A2E + NFD exhibited a decreased level of Ca^2+^ when compared with the control cell (*P* < 0.05). We also observed that the Ca^2+^ level in the BL + NFD, A2E, and A2E + NFD-treated cells was significantly decreased when compared with BL-treated cells (*P* < 0.05). Finally, the results revealed lower levels of Ca^2+^ in the A2E + BL and A2E + BL + NFD-treated cells than in the BL + NFD-treated RPE (*P* < 0.05) and higher levels in cells treated with A2E + BL + NFD than A2E (*P* < 0.05) (Figure 4).

### 3.5. Mitochondrial Membrane Potential (ΔΨ*m*)

**Figure 5:**
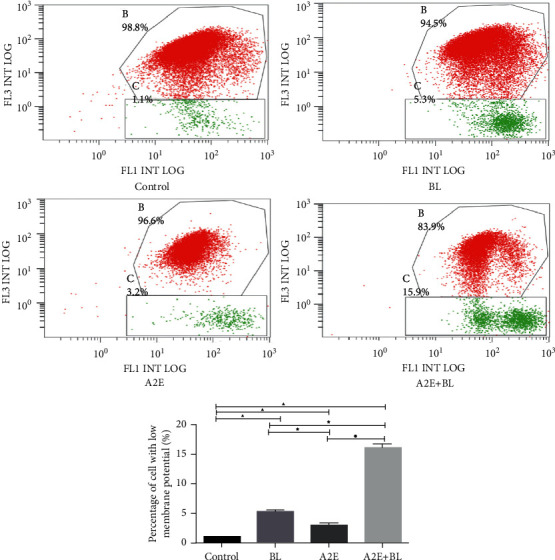
Mitochondrial membrane potential. The mitochondrial membrane potential in the indicated groups was analyzed by flow cytometry with JC-1-stained RPE cells. The decrease in the mitochondrial membrane potential was detected by the transition of JC-1 red fluorescence (FL-3) to JC-1 green fluorescence (FL-1). Statistical analysis of RPE cells with low Δѱ*m* (%) (the percentage of the cells in C). Data are shown as the mean ± SD (*n* = 3). An LSD test following one-way ANOVA was performed. Compared to the control group, ▲*P* < 0.05; compared to the BL group, ★*P* < 0.05; compared to the BL + NFD group, ●*P* < 0.05; and compared to the A2E group, ◇*P* < 0.05.

The decrease in mitochondrial membrane potential is a notable event in the early stages of apoptosis. When the mitochondrial membrane potential is high, JC-1 accumulates in the mitochondrial matrix to form a polymer, which fluoresces red, and when the mitochondrial membrane potential is low, JC-1 remains a monomer, which fluoresces green. Compared with those in the control (low ΔΨ*m*, 1.07%), the BL (low ΔΨ*m*, 5.37%), A2E (low ΔΨ*m*, 3.00%), and BL + A2E-treated (low ΔΨ*m*, 16.00%) cells had a decreased mitochondrial membrane potential (*P* < 0.05) (Figure 5).

## 4. Discussion

AMD is a degenerative retinal disease that causes irreversible vision loss [13]. The etiology of AMD includes age, smoking, genetic factors, and chronic light damage, including that from sunlight [14, 15]. Among the wavelengths in the visible light spectrum, BL waves have a higher association with the induction of AMD, due to the increased levels of energy reaching the macula, inducing structural and functional alterations in RPE cells, as well as retinal phototoxicity [16, 17]. The accumulation of lipofuscin in the retinal pigment epithelium is proportional to eye aging and is related to various forms of retinal degeneration. In presence of irradiation, the ability of lipofuscin to produce superoxide radicals is significantly enhanced [18, 19]. Moreover, A2E, the main fluorophore in lipofuscin, can upregulate the expression of inflammatory factors and vascular endothelial growth factor [20, 21]. Upon accumulation or light exposure, A2E can damage RPE cells [22]. When A2E is exposed to BL, it can be oxidized into peroxide-A2E and furan-A2E, which leads to oxidative stress, inflammatory response, as well as the apoptosis of RPE cells.

Ca^2+^, as a second messenger, controls many cellular processes, including cell differentiation, signaling, proliferation, migration, and death [23, 24]. The cytoplasmic Ca^2+^ concentration in the cell resting state is very low, approximately 10^−7^ mol/L [25]. In this study, the Ca^2+^ level in the cytoplasm increased after BL irradiation and increased further after A2E was added to the culture, before exposure to BL, indicating that A2E and BL have synergistic effects. On the one hand, the activation of calcium channels by A2E and BL on the plasma membrane leads to extracellular Ca^2+^ influx [6, 26], and on the other hand, inositol 1, 4, 5-triphosphate (IP3) receptors mediate the release of Ca^2+^ from the endoplasmic reticulum, which increases the level of Ca^2+^ in the cytoplasm and directly stimulates mitochondrial Ca^2+^ uptake [27, 28]. When the L-type calcium channel blocker NFD was added to the cells, the Ca^2+^ level in the cytoplasm decreased, indicating that NFD can inhibit the influx of extracellular Ca^2+^ and protect the cells. When cytosolic Ca^2+^ levels increase, excess Ca^2+^ can bind to calcium-dependent proteases, which in turn activates the downstream Bcl-2 family and triggers the mitochondrial apoptosis pathway. After the cell is exposed to external stimuli, G protein-coupled receptors activate phospholipase C, which hydrolyzes membrane phosphatidylinositol 4,5-diphosphate (PIP2) to IP3 and diacylglycerol (DAG). IP3R mediates the release of Ca^2+^ from the endoplasmic reticulum and can directly stimulate the mitochondrial absorption of Ca^2+^ [27, 28], while PKC phosphorylates various proteins and enzymes that can induce apoptosis [29].

Lysosomes are intracellular acid organelles and were recently shown to be the second-largest calcium reservoir [30]. The endoplasmic reticulum is the main source of Ca^2+^ in lysosomes, and membrane contact sites found between the endoplasmic reticulum and lysosomes play a crucial role in the lysosomal Ca^2+^ refilling [31, 32]. In this experiment, the level of Ca^2+^ in lysosomes increased after illumination, which may be related to the enhanced Ca^2+^ release upon endoplasmic reticulum stress; this phenomenon, in turn, increases Ca^2+^ uptake by lysosomes to maintain intracellular calcium homeostasis. To internalize Ca^2+^, lysosomes need to overcome the pH gradient; however, much is unknown about how lysosomes take up Ca^2+^, and there may be lysosomal Ca^2+^ transporters similar to those in mitochondria. When the cells were loaded with A2E and then illuminated, the Ca^2+^ level in the lysosomes decreased. This phenomenon may be attributed to A2E damaging the lysosomal membrane and leading to the leakage of Ca^2+^ from lysosomes. A2E is a photosensitizer that generates free radicals with radiation, inducing a decrease in the lysosomal membrane integrity, with consequent release of lysosomal molecules (including enzymes and Ca^2+^) into the cytoplasm. Thus, A2E also increases the pH in lysosomes, which may hinder pH-dependent activities and block Ca^2+^ uptake by lysosomes [9, 33].

The mitochondrial membrane potential is necessary to synthesize ATP, and a decrease in membrane potential is a characteristic event in early apoptosis [34]. Measurements of the mitochondrial membrane potential in human RPE cells revealed that both BL and A2E reduced the mitochondrial membrane potential and had a synergistic effect. RPE cells produce a large amount of ROS under BL and A2E stress, and these ROS may damage the mitochondrial membrane structure, resulting in a decrease in membrane potential and mitochondrial dysfunction [35, 36]. In addition, it was described that 25 *μ*M A2E-laden cells under both dark and light conditions exhibited a fragmented mitochondrial network, a feature from dysfunctional mitochondria, which could ultimately trigger apoptotic cell death [37].

Mitochondria and the endoplasmic reticulum are closely connected and form dynamic platforms called mitochondrial-associated endoplasmic reticulum membranes (MAMs), which facilitate the transfer of Ca^2+^ from the endoplasmic reticulum to mitochondria [38]. In this study, BL increased the Ca^2+^ level in mitochondria, but exposure to light after A2E treatment led to a reduced Ca^2+^ level. This finding may be due to the release of Ca^2+^ from the endoplasmic reticulum after BL irradiation, which increases the cytosolic Ca^2+^ level. The internalization of Ca^2+^ by mitochondria increases to prevent an excessive increase in cytosolic Ca^2+^. In this case, the uptake of Ca^2+^ by mitochondria is considered a defense mechanism that plays an important role in protecting cells [39]. However, when mitochondria are overloaded with Ca^2+^ to the point that their own tolerance limits are exceeded, Ca^2+^ causes substantial changes in mitochondrial function and irreversible damage, including defects to the mitochondrial electron transport chain (ETC), reduced ATP production, and increased ROS production [40]. Ca^2+^ and ROS are the most important drivers inducing the opening of mitochondrial permeability transition pores (MPTPs). The opening of MPTPs can cause the mitochondrial transmembrane potential to disappear, leading to the release of accumulated Ca^2+^, cytoplasmic Ca^2+^ overload, release of mitochondrial proteins such as cytochrome C and AIF into the cytoplasm, and the activation of caspase-9 and caspase-3, which induces cell death [34, 41]. NFD can inhibit Ca^2+^ influx and attenuate the oxidative stress response.

The present study has several limitations. Since there are no simple A2E-treated cells, it is not possible to explore the effect of A2E alone on intracellular Ca^2+^ level. For the detection of Ca^2+^ level in lysosomes, because the Ca^2+^ fluorescence probe in cells is sensitive to pH, other detection methods can be explored in the later stage to obtain more accurate results.

## 5. Conclusion

The present study reveals that upon blue light irradiation, intracellular Ca^2+^ levels increase and the mitochondrial membrane potential decreases. Our findings suggest that mitochondria and lysosomes can internalize excessive cytosolic Ca^2+^ to prevent cell damage. However, when the cells were loaded with A2E and then exposed to light, due to the loss of membrane integrity, the Ca^2+^ and other molecules involved in apoptosis are released from these organelles inducing the activation of several pathways involved in cellular death. In this process, both blue light and A2E can reduce the mitochondrial membrane potential, and they have a synergistic effect. However, since intracellular calcium fluorescence probes are sensitive to pH, other detection methods to measure Ca^2+^ levels in lysosomes should be explored in the future to obtain more accurate results, which may help elucidate human RPE cell apoptosis induced by blue light.

## Data Availability

The data that support the findings of this study are available from the corresponding author (Shan-Jun Cai) upon reasonable request.

## Abbreviations

A2E: N-Retinylidene-N-retinylethanolamine
RPE: Retinal pigment epithelial
AMD: Age-related macular degeneration
ROS: Reactive oxygen species.

## References

[1] Crouch R. K., Koutalos Y., Kono M., Schey K., Ablonczy Z. (2015). A2E and lipofuscin. *Progress in Molecular Biology and Translational Science*.

[2] Höhn A., Grune T. (2013). Lipofuscin: formation, effects and role of macroautophagy. *Redox Biology*.

[3] Takeshima H., Venturi E., Sitsapesan R. (2015). New and notable ion-channels in the sarcoplasmic/endoplasmic reticulum: do they support the process of intracellular Ca2+release?. *The Journal of Physiology*.

[4] Hammond B. R., Sreenivasan V., Suryakumar R. (2019). The effects of blue light-filtering intraocular lenses on the protection and function of the visual system. *Clinical Ophthalmology*.

[5] Nita M., Grzybowski A. (2016). The role of the reactive oxygen species and oxidative stress in the pathomechanism of the age-related ocular diseases and other pathologies of the anterior and posterior eye segments in adults. *Oxidative Medicine and Cellular Longevity*.

[6] Shamsi F. A., Boulton M. (2001). Inhibition of RPE lysosomal and antioxidant activity by the age pigment lipofuscin. *Investigative Ophthalmology & Visual Science*.

[7] Brailoiu G. C., Brailoiu E. (2016). Modulation of calcium entry by the endo-lysosomal system. *Advances in Experimental Medicine & Biology*.

[8] Brini M., Calì T., Ottolini D., Carafoli E. (2013). The plasma membrane calcium pump in health and disease. *FEBS Journal*.

[9] López-Sanjurjo C. I., Tovey S. C., Prole D. L., Taylor C. W. (2013). Lysosomes shape Ins(1,4,5)P3-evoked Ca^2+^ signals by selectively sequestering Ca^2+^ released from the endoplasmic reticulum. *Journal of Cell Science*.

[10] Su G., Cai S. J., Gong X., Wang L. L., Li H. H., Wang L. M. (2016). Establishment of a blue light damage model of human retinal pigment epithelial cells in vitro. *Genetics and Molecular Research*.

[11] Feng J., Chen X., Sun X. (2014). Expression of endoplasmic reticulum stress markers GRP78 and CHOP induced by oxidative stress in blue light-mediated damage of A2E-containing retinal pigment epithelium cells. *Ophthalmic Research*.

[12] Lu B., Zhang P., Zhou M. (2017). Involvement of XBP1s in blue light-induced A2E-containing retinal pigment epithelium cell death. *Ophthalmic Research*.

[13] Nowak J. Z. (2013). Oxidative stress, polyunsaturated fatty acids-derived oxidation products and bisretinoids as potential inducers of CNS diseases: focus on age-related macular degeneration. *Pharmacological Reports*.

[14] Sui G. Y., Liu G. C., Liu G. Y. (2013). Is sunlight exposure a risk factor for age-related macular degeneration? A systematic review and meta-analysis. *British Journal of Ophthalmology*.

[15] Schick T., Ersoy L., Lechanteur Y. T. (2016). History of sunlight exposure is a risk factor for age-related macular degeneration. *Retina*.

[16] Mainster M. A., Turner P. L. (2010). Blue-blocking IOLs decrease photoreception without providing significant photoprotection. *Survey of Ophthalmology*.

[17] Loane E., Kelliher C., Beatty S., Nolan J. M. (2008). The rationale and evidence base for a protective role of macular pigment in age-related maculopathy. *British Journal of Ophthalmology*.

[18] Rózanowska M., Pawlak A., Rózanowski B. (2004). Age-related changes in the photoreactivity of retinal lipofuscin granules: role of chloroform-insoluble components. *Investigative Ophthalmology & Visual Science*.

[19] Gaillard E. R., Atherton S. J., Eldred G., Dillon J. (1995). Photophysical studies on human retinal lipofuscin. *Photochemistry and Photobiology*.

[20] Blasiak J. (2020). Senescence in the pathogenesis of age-related macular degeneration. *Cellular and Molecular Life Sciences*.

[21] Parmar V. M., Parmar T., Arai E., Perusek L., Maeda A. (2018). A2E-associated cell death and inflammation in retinal pigmented epithelial cells from human induced pluripotent stem cells. *Stem Cell Research*.

[22] Yoon S. M., Lee B. L., Guo Y. R., Choung S. Y. (2016). Preventive effect of *Vaccinium uliginosum* L. extract and its fractions on age-related macular degeneration and its action mechanisms. *Archives of Pharmacal Research*.

[23] Cheng Z. Y., Wang X. P., Schmid K. L., Han X. G., Song H., Tang X. (2015). GABA_A*α*1_ and GABA_A*ρ*1_ subunits are expressed in cultured human RPE cells and GABA_A_ receptor agents modify the intracellular calcium concentration. *Molecular Vision*.

[24] Schwaller B. (2010). Cytosolic Ca^2+^ buffers. *Cold Spring Harbor Perspectives in Biology*.

[25] Raffaello A., Mammucari C., Gherardi G., Rizzuto R. (2016). Calcium at the center of cell signaling: interplay between endoplasmic reticulum, mitochondria, and lysosomes. *Trends in Biochemical Sciences*.

[26] Roderick H. L., Berridge M. J., Bootman M. D. (2003). Calcium-induced calcium release. *Current Biology*.

[27] Cui C., Merritt R., Fu L., Pan Z. (2017). Targeting calcium signaling in cancer therapy. *Acta Pharmaceutica Sinica B*.

[28] Dickson E. J., Falkenburger B. H., Hille B. (2013). Quantitative properties and receptor reserve of the IP(3) and calcium branch of G(q)-coupled receptor signaling. *The Journal of General Physiology*.

[29] Xiong W. H., Pang J. J., Pennesi M. E., Duvoisin R. M., Wu S. M., Morgans C. W. (2015). The effect of PKC*α* on the light response of rod bipolar cells in the mouse retina. *Investigative Ophthalmology & Visual Science*.

[30] Xu H., Ren D. (2015). Lysosomal physiology. *Annual Review of Physiology*.

[31] Kilpatrick B. S., Eden E. R., Schapira A. H., Futter C. E., Patel S. (2013). Direct mobilisation of lysosomal Ca^2+^ triggers complex Ca^2+^ signals. *Journal of Cell Science*.

[32] Eden E. R., White I. J., Tsapara A., Futter C. E. (2010). Membrane contacts between endosomes and ER provide sites for PTP1B-epidermal growth factor receptor interaction. *Nature Cell Biology*.

[33] Bermann M., Schütt F., Holz F. G., Kopitz J. (2001). Does A2E, a retinoid component of lipofuscin and inhibitor of lysosomal degradative functions, directly affect the activity of lysosomal hydrolases?. *Experimental Eye Research*.

[34] Li X., Fang F., Gao Y. (2019). ROS induced by killerred targeting mitochondria (mtKR) enhances apoptosis caused by radiation via cyt c/caspase-3 pathway. *Oxidative Medicine and Cellular Longevity*.

[35] Mélanie M., Karine B., Claire A. (2018). Light action spectrum on oxidative stress and mitochondrial damage in A2E-loaded retinal pigment epithelium cells. *Cell Death & Disease*.

[36] Nakanishi-Ueda T., Majima H. J., Watanabe K. (2013). Blue LED light exposure develops intracellular reactive oxygen species, lipid peroxidation, and subsequent cellular injuries in cultured bovine retinal pigment epithelial cells. *Free Radical Research*.

[37] Agustina A., Guadalupe G. L., Juan M. B. (2019). Toxicity of blue led light and A2E is associated to mitochondrial dynamics impairment in ARPE-19 cells: implications for age-related macular degeneration. *Archives of Toxicology*.

[38] Missiroli S., Patergnani S., Caroccia N. (2018). Mitochondria-associated membranes (MAMs) and inflammation. *Cell Death & Disease*.

[39] Badone B., Ronchi C., Kotta M. C. (2018). Calmodulinopathy: functional effects of CALM mutations and their relationship with clinical phenotypes. *Frontiers in Cardiovascular Medicine*.

[40] Rimessi A., Previati M., Nigro F., Wieckowski M. R., Pinton P. (2016). Mitochondrial reactive oxygen species and inflammation: molecular mechanisms, diseases and promising therapies. *The International Journal of Biochemistry & Cell Biology*.

[41] Tajeddine N. (2016). How do reactive oxygen species and calcium trigger mitochondrial membrane permeabilisation?. *Biochimica et Biophysica Acta*.

